# Supplementary material for: The Clinical Utility of Selected Coagulation Parameters in Predicting the Risk of Venous Thromboembolism in Neuroendocrine Tumours: A Prospective, Single-Centre Study
Source: Cancers (Basel). 2025 Oct 22;17(21):3405. doi: 10.3390/cancers17213405 (PMC12610385; doi:10.3390/cancers17213405)
Supplement: Supplementary file 1 [file cancers-17-03405-s001.zip › Supplementary Figure S1.pdf]

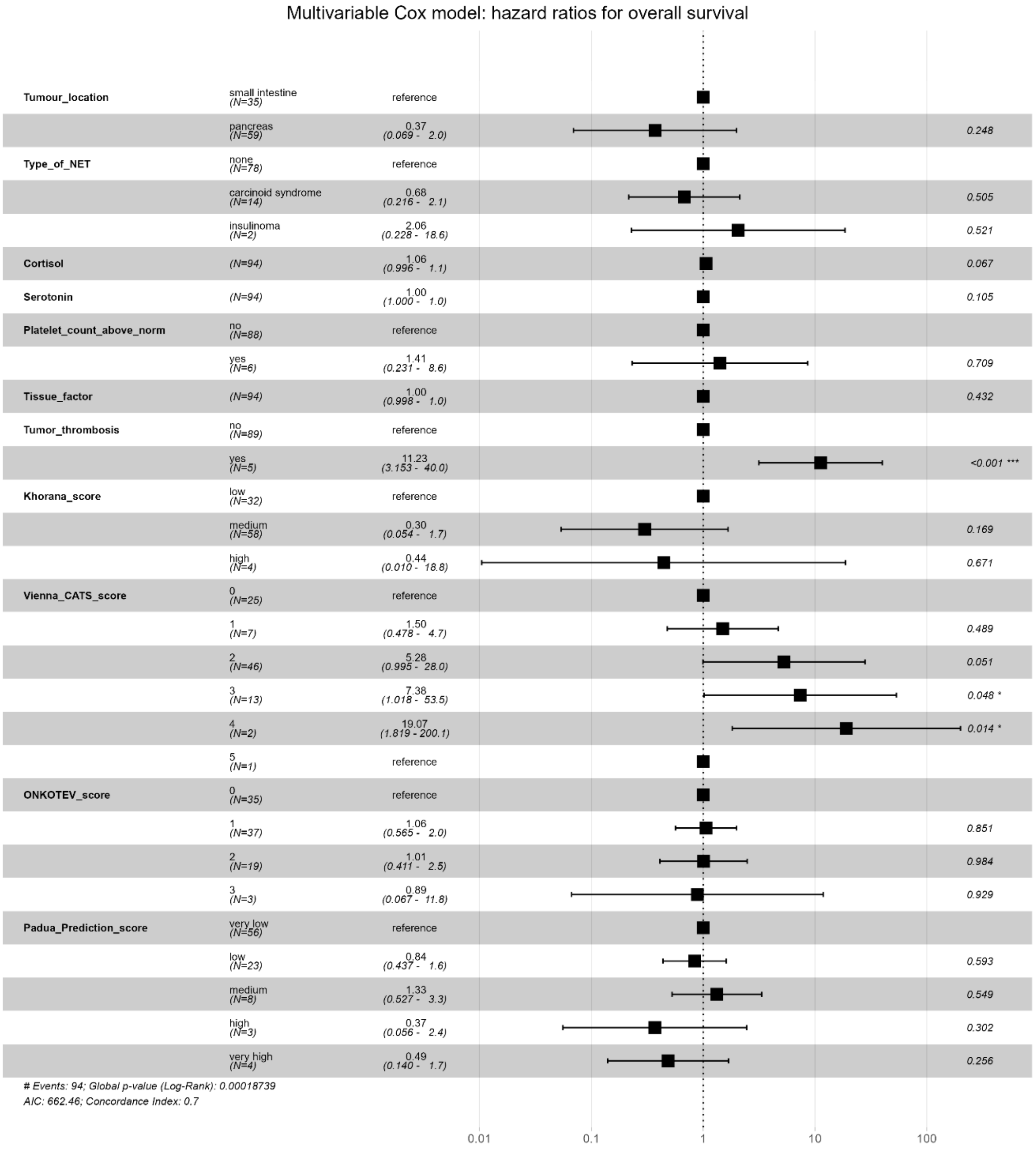

Supplementary Figure S1. Multivariate analysis of selected parameters without categorization of cortisol, serotonin and tissue factor, and VTE risk assessment scales in the Cox regression model. Presence of TT ($p < 0.001$), and Vienna CATS score (score = 3, $p = 0.048$, score = 4, $p = 0.014$) were statistically significant in the specific Cox model results. Model concordance index = 0.7, model $p < 0.001$.
